# Nanoporous Materials as New Engineered Catalysts for the Synthesis of Green Fuels

**DOI:** 10.3390/molecules20045638

**Published:** 2015-03-31

**Authors:** Ioana Fechete, Jacques C. Vedrine

**Affiliations:** 1 Institut de Chimie et Procédés pour l’Energie, l’Environnement et la Santé—ICPEES, UMR 7515 CNRS, Université de Strasbourg, 25 rue Becquerel, 67087 Strasbourg Cedex 2, France; 2 Laboratoire de Réactivité de Surface, UMR-CNRS 7197, Université P. & M. Curie-Paris 06, Sorbonne Universités, 4 Place Jussieu, 75252 Paris, France

**Keywords:** zeolites, mesoporous materials, methane, Synthetic Natural Gas (SNG), Ni nanoparticles, deactivation prevention

## Abstract

This review summarizes the importance of nanoporous materials and their fascinating structural properties with respect to the catalytic and photocatalytic reduction of CO_2_ to methane, toward achieving a sustainable energy supply. The importance of catalysis as a bridge step for advanced energy systems and the associated environmental issues are stressed. A deep understanding of the fundamentals of these nanoporous solids is necessary to improve the design and efficiency of CO_2_ methanation. The role of the support dominates the design in terms of developing an efficient methanation catalyst, specifically with respect to ensuring enhanced metal dispersion and a long catalyst lifetime. Nanoporous materials provide the best supports for Ni, Ru, Rh, Co, Fe particles because they can prevent sintering and deactivation through coking, which otherwise blocks the metal surface as carbon accumulates. This review concludes with the major challenges facing the CO_2_ methanation by nanoporous materials for fuel applications.

## 1. Introduction

Energy generated via renewable energy strategies is critical to various aspects of global human development, including harmony, equity, employment, ecosystems, and environmental protection. As the demand for energy continues to increase worldwide, mainly in emerging economies, overcoming energy barriers is a key step in the continued development of civilization. Modern economic development (chemical industry, power plants, transportation sector) is inherently dependent on fossil fuels, which are non-renewable energy sources [1,2,3,4,5], yet fossil fuels still represent more than 85% of the world energy consumption. Since the industrial revolution in the 19th century, the majority of anthropogenic CO_2_ emissions have been attributed to the consumption of fossil fuels [6,7,8,9,10,11,12], resulting in environmental pollution and very probably in increased global warming effect. In this context, a challenge for 21st century is the control of technological processes, developing highly active and selective reaction systems with atom efficiency and minimizing unwanted secondary reactions [13,14,15,16,17,18,19,20,21,22]. Excessive CO_2_ emissions have caused global climate change and increased planetary temperatures; more specifically, the global surface temperature has increased by 0.74 °C over the past century. The CO_2_ levels in the earth atmosphere have increased significantly by more than 10 billion tonnes per year. Moreover, heavy dependence on fossil fuels causes problems in energy security because a large fraction of the fossil fuel consumed is imported, and these resources are non-renewable. Therefore, sustainable energy production, combined with moderate consumption practices, represents a challenge to our civilization [1,2,4,5,23,24,25]. Alternative energy sources should be derived from geothermal, hydrothermal, solar, wind, nuclear and other renewable resources. One perspective is that all sectors should employ low-pollution renewable energy with the net “zero emission” of CO_2_. However, several petrochemical companies are considering biomass and natural gas fuel sources as alternative renewable feedstocks.

**Figure 1:**
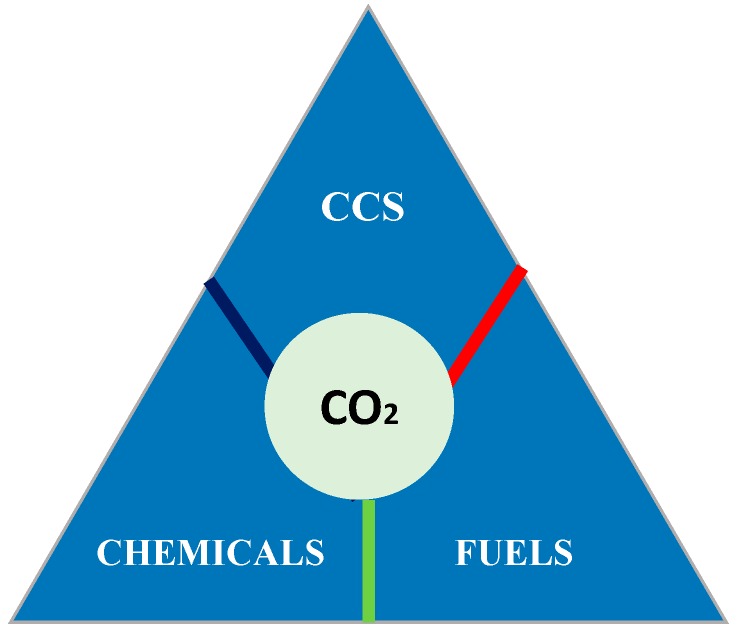
Possible strategies of reducing CO_2_.

Several strategies should be considered to reduce the amount of carbon dioxide emitted into the atmosphere. Examples include the implementation of new green technologies by exchanging the fossil fuels for renewable energy sources, the biological, physical and chemical capture and storage of CO_2_, and the conversion of CO_2_ to various chemical products and fuels as shown in Figure 1 [2,4,11,12,21,24,26].

Carbon capture and storage (CCS) has received major attention in recent years [27,28,29,30,31,32]. In this strategy, several approaches have been tested for the removal of CO_2_ from power plants or other large emitters. These approaches include amine scrubbing, cryogenic distillation, membrane separation and the use of an absorbent/adsorbent [33]. Amine scrubbing is inconvenient in that this approach is inherently corrosive and there exists difficulty in solvent regeneration. In comparison, cryogenic distillation imposes a high energetic demand. Membrane separation is limited by permeability-selectivity. In contrast, the adsorption of CO_2_ into nanoporous materials is both an energy efficient process and offers separation capability. Several nanoporous adsorbents have been tested for CO_2_ capture [33]. Among these nanoporous materials, the zeolites and MOF have predominantly been studied, both of which present high selectivities for the separation of flue gas. The high selectivity of these materials has been explained by the zeolite electric field, which inherently varies in strength, further suggesting that the architecture of the zeolite structure is a very important feature [34,35,36]. These nanoporous materials have been suggested as suitable materials for CO_2_/CH_4_ separation. In the CCS approach, CO_2_ is captured in either a pre- or post-combustion process. In pre-combustion capture, the partial oxidation of a feedstock fuel produces syngas, which reacts with steam to yield shifted syngas. In the post-combustion capture process, the CO_2_ is separated from the exhaust flue gas, which primarily consists of N_2_ and CO_2_ [34,35,36]. The captured CO_2_ is then stored either in the deep ocean or underground in geological formations such as depleted oil and gas reservoirs. The problem there is that CO_2_ should be inert chemically with respect to the geological rocks, in presence of water and for a very long time. Although the implementation of CSS will lead to decreased emissions of CO_2_ into the atmosphere and will yield raw materials, implementation with respect to the world economy is an inconvenient aspect of this approach. For CCS, the sequestration site must be near the CO_2_ source. Often, the CO_2_ must eventually be transported to a different site, which necessitates further investment for the development of adequate infrastructure. A plausible alternative CCS strategy is the development of a new catalytic process or improvements to those already in existence for the use of captured carbon as a reagent to produce useful chemicals or fuel.

The conversion of CO_2_ should be used as an alternative to petrochemistry and petrorefineries and as a bridging technology toward the development of a sustainable energy supply, and consequently sustainable industrial development, for the long term [37]. However, the process of CO_2_ valorization by conversion into chemical products has been known for many decades; more specifically, the synthesis of salicylic acid was discovered in 1969 [2,38], the synthesis of NaHCO_3_–Na_2_CO_3_ was developed in 1882 and was known as the “Solvay process” [39] and the synthesis of urea was identified in 1922 [40]. In 1970 [2], the catalytic conversion of CO_2_ to synthesize methanol from syngas enriched with CO_2_ was first developed. The valorization of CO_2_ by conversion into other chemicals has been discussed in several reviews [2,3,11,12,24]. A promising approach for the valorization of CO_2_ was the catalytic conversion of CO_2_ into fuels: Fischer-Tropsch, MeOH, DME, CH_4_ (synthetic natural gas—SNG), and syngas.

In this review, we focus only on CO_2_ methanation for the SNG process. Methanation involves the catalytic hydrogenation of carbon oxides to provide an efficient alternative to conventional natural gas [41]; moreover, the infrastructure for handling CH_4_ is well established. SNG is considered a promising approach for obtaining this valuable, high-combustion-efficiency gaseous fuel which can be used in current hydrocarbon-based automobiles and transportation systems while reducing dependence on petroleum; in addition, this fuel is considered environmentally friendly as it recovers CO_2_ used as reactant in its synthesis [42,43,44,45,46]. Catalysis is a core technology of the energy industry [47,48,49,50,51] with an important role in the area production of sustainable fuels and energy [4,5,52,53,54,55,56,57,58]. The development of suitable catalysts for the conversion of CO_2_ is under intensive study by researchers around the world. A large number of excellent studies on various metal catalysts have appeared in the literature [59,60,61,62,63], and supported nickel catalysts are the most widely studied for CO_2_ methanation due to their high ability to dissociate CO_2_ [64,65]. Their higher activities and selectivities for methane have been attributed to various factors, including the nature of the support, nature of the metal, amount of metal and its dispersion. However, these traditional catalysts suffer from several drawbacks, including sensitivity to metal site poisoning, sintering, coke deposition, and deactivation [66,67,68,69,70]. In light of the ubiquitous restrictions imposed by environmental legislation and economics, the use of nanostructured porous catalysts, which can be very selective for the desired products, is one alternative to the use of traditional catalysts [71,72]. Nanoporous catalysts are of great interest because of their highly ordered pore structure, high specific surface area, and tailorable pore size, framework, and surface properties [73,74,75,76,77,78,79,80,81,82]. Nanoporous materials can be used as hosts for the preparation of nano-sized catalysts. The advantages of the small metal particles of nanoporous catalyst are a great variety in the valence band electron structure, short range ordering, enhanced interaction with the environment due to the high number of dangling bonds, and self-structuring for optimum performance in chemisorption. Surface area is the main factor which controls the catalytic activity of a nanoporous catalyst. The surface area of a porous material is higher than the surface of a non-porous material. The large surface area leads to a well-distributed dispersion of the catalytic phase at high loadings; this can hardly be achieved with non-porous traditional support, such as silica gel or alumina. The uniform porosity results in monodispersed nanometer-sized catalysts. In cases where catalytic activity is size-sensitive, it is very desirable to use nanochannels as a confinement zone to obtain the nanocatalyst. Taking into account that the catalytic reactions occur on surface of the catalysts, the higher surface leads to an improved activity and selectivity. One of crucial points is whether turnover frequency measured for a given catalytic reaction increases or decreases as the particle size is diminished.

Nanoporous catalysts play an important role in all areas of catalysis, especially in energy and environmental applications [83,84,85,86,87,88,89,90,91]. The structural aspects of nanoporous materials have been summarized in several excellent publications [73,92,93,94,95,96,97,98]. Therefore, a meaningful assessment of the importance of nanoporous catalysts over time should be performed. This review comprehensively discusses the catalytic and photocatalytic conversion of CO_2_ to CH_4_ and the role of nanoporous catalysts in these reactions.

## 2. Thermodynamics of CO_2_

CO_2_ is kinetically and thermodynamically stable and the methanation reaction is extremely exothermic due to the high concentration of oxidized carbon forms in the feed gas [99]. The chemical reactions are driven by the difference between the Gibbs free energy values of the reactants and products of the chemical reaction, as shown by the Gibbs–Helmholtz equation: ΔG° = ΔH° − TΔS°. A large energy input, optimized reaction conditions and active catalysts are necessary to transform CO_2_ into useful products. This is because CO_2_ is inert: the carbon atom in CO_2_ is in its most oxidized state, which means that its chemical transformation is thermodynamically highly unfavorable [100]. In this case, as a raw material, CO_2_ is in its lowest energy state, constituting a major obstacle in the establishment of industrial processes for CO_2_ conversion.

Upon analyzing the Gibbs free energy of the exothermic hydrogenation of CO_2_, a majority of the associated reactions are found to be thermodynamically unfavorable. Because the Gibbs free energy values are more positive than the corresponding ΔH° values, they are less favorable; only a few reactions have both negative ΔG° and ΔH° values. Values of ΔG < 0 correspond to hydrogenation or reactions with products containing C-O bonds. Favorable values of ΔG in the hydrogenation reaction are associated with the formation of water. Because hydrogen must be produced at the cost of the input energy, none of these reactions is favorable for CO_2_ mitigation.

The values of the enthalpy and Gibbs free energy, calculated by ASPEN software [3,101,102,103], of the exothermic, ΔH < 0, reaction in the CO_2_ hydrogenations are as follows:

CO_2(g)_ + H_2(g)_ → HCOOH_(l)_; ΔH° = −31.0 kJ·mol^−1^; ΔG° = +34.3 kJ·mol^−1^ (1)

CO_2(g)_ + 2H_2(g)_ → HCHO_(g)_ + H_2_O_(l)_; ΔH ° = −11.7 kJ·mol^−1^; ΔG° = +46.6 kJ·mol^−1^ (2)

CO_2(g)_ + 3H_2(g)_ → CH_3_OH_(l)_ + H_2_O_(l)_; ΔH° = −137.8 kJ·mol^−1^; ΔG° = −10.7 kJ·mol^−1^ (3)

CO_2(g)_ + 4H_2(g)_ → CH_4(g)_ + 2H_2_O_(l)_; ΔH° = −259.9 kJ·mol^−1^; ΔG° = −132.4 kJ·mol^−1^ (4)

2CO_2(g)_ + H_2(g)_ → (COOH)_2(l)_; ΔH° = −39.3 kJ·mol^−1^; ΔG° = 85.3 kJ·mol^−1^ (5)

2CO_2(g)_ + 6H_2(g)_ → CH_3_OCH_3(g)_ + 3H_2_O_(l)_; ΔH° = −264.9 kJ·mol^−1^; ΔG° = −38.0 kJ·mol^−1^ (6)

CO_2(g)_ + H_2_ + CH_3_OH_(l)_ → HCOOCH_3(l)_ + H_2_O_(l)_; ΔH° = −31.8 kJ·mol^−1^; ΔG° = −25.8·kJ mol^−1^ (7)

CO_2(g)_ + H_2_ + CH_3_OH_(l)_ →CH_3_COOH_(l)_ + H_2_O_(l)_; ΔH° = −135.4 kJ·mol^−1^; ΔG° = −63.6 kJ·mol^−1^ (8)

CO_2(g)_ + 3H_2(g)_ + CH_3_OH_(l)_ → C_2_H_5_OH_(l)_ + 2H_2_O_(l)_; ΔH° = −221.6 kJ·mol^−1^ ; ΔG° = −88.9 kJ·mol^−1^ (9)

CO_2(g)_ + H_2(g)_ + NH_3(g)_ → HCONH_2(l)_ + H_2_O_(l)_; ΔH° = −103.0 kJ·mol^−1^; ΔG° = +7.2 kJ·mol^−1^ (10)

CO_2(g)_ + CH_4(g)_ → CH_3_COOH_(l)_; ΔH° = −13.3 kJ·mol^−1^; ΔG° = +58.1 kJ·mol^−1^ (11)

CO_2(g)_ + CH_4(g)_ + H_2(g)_ → CH_3_CHO_(l)_ + H_2_O_(l)_; ΔH° = −14.6 kJ·mol^−1^; ΔG° = +74.4 kJ·mol^−1^ (12)

CO_2(g)_ + CH_4(g)_ + 2CO_2(g)_ → (CH_3_)_2_CO_(l)_ + H_2_O_(l)_; ΔH° = −70.5 kJ·mol^−1^; ΔG° = +51.2 kJ·mol^−1^ (13)

CO_2(g)_ + C_2_H_2(g)_ + H_2(g)_ → CH_2_ = CHCOOH_(l)_; ΔH° = −223.6 kJ·mol^−1^; ΔG° = −115.0 kJ·mol^−1^ (14)

CO_2(g)_ + C_2_H_4(g)_ → CH_2_ = CHCOOH_(l)_; ΔH° = −49.1 kJ·mol^−1^; ΔG° = +26.2 kJ·mol^−1^ (15)

CO_2(g)_ + C_2_H_4(g)_ + H_2(g)_ → C_2_H_5_COOH_(l)_; ΔH° = −166.6 kJ·mol^−1^; ΔG° = −56.6 kJ·mol^−1^ (16)

CO_2(g)_ + C_2_H_4(g)_ + 2H_2(g)_ → C_2_H_5_CHO + H_2_O_(l)_; ΔH° = −171.1 kJ·mol^−1^; ΔG° = −44.4 kJ·mol^−1^ (17)

CO_2(g)_ + C_6_H_6(l)_ → C_6_H_5_COOH_(l)_; ΔH° = −21.6 kJ·mol^−1^; ΔG° = +30.5 kJ·mol^−1^ (18)

CO_2(g)_ + C_6_H_5_OH_(l)_ → mC_6_H_4_(OH)COOH_(l)_; ΔH° = −6.6 kJ·mol^−1^; ΔG° = +46.9 kJ·mol^−1^ (19)

Hydrogenation reactions of CO_2_ with ΔH > 0 can be performed [3,101,102,103]. These reactions are associated with highly positive ΔG° values and are not favorable.

CO_2(g)_ + CH_2_=CH_2(g)_ → CH_2_CH_2_O_(l)_ + CO_(g)_; ΔH° = +152.9 kJ·mol^−1^; ΔG° = +177.3 kJ·mol^−1^ (20)

CO_2(g)_ + C(s) →2CO_(g)_; ΔH° = +172.6 kJ·mol^−1^; ΔG° = +119.9 kJ·mol^−1^ (21)

3CO_2(g)_ CH_4(g)_ → 4CO_(g)_ + 2H_2_O_(l)_; ΔH° = +235.1 kJ·mol^−1^; ΔG° = +209.2 kJ·mol^−1^ (22)

CO_2(g)_ + CH_4(g)_ → 2CO_(g)_ + 2H_2(g)_; ΔH° = +247.5 kJ·mol^−1^; ΔG° = +170.8 kJ·mol^−1^ (23)

CO_2(g)_ + 2CH_4(g)_ → C_2_H_6(g)_ + CO_(g)_ + H_2_O_(l)_; ΔH° = +58.8 kJ·mol^−1^; ΔG° = +88.0 kJ·mol^−1^ (24)

2CO_2(g)_ + 2CH_4(g)_ → C_2_H_4(g)_ + 2CO_(g)_ + 2H_2_O_(l)_; ΔH° = +189.7 kJ·mol^−1^; ΔG° = +208.3 kJ·mol^−1^ (25)

CO_2(g)_ + C_2_H_4(g)_ → C_2_H_4_O_(g)_ + CO_(g)_; ΔH° = +178.0 kJ·mol^−1^; ΔG° = +176.0 kJ·mol^−1^ (26)

In general, the conversion of CO_2_ is accompanied by the production of CO. The reaction enthalpies for the production of the same product from either CO or CO_2_ are comparable, although in most cases, CO is favored compared to CO_2_ [3,101,102,103].

## 3. Reaction Mechanism of CO_2_ Methanation

**Figure 2:**
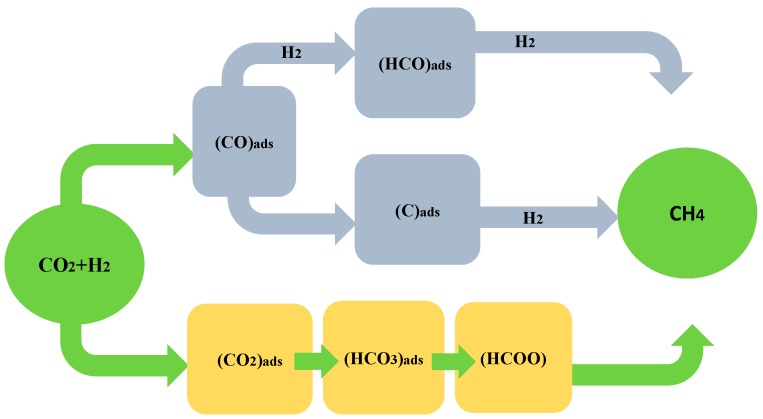
Reaction mechanisms of CO_2_ methanation.

Over the past few decades, understanding the mechanism of the methanation of CO_2_ has represented a significant challenge. Following a careful analysis of several studies, we ascertained that in the methanation of CO_2_, two types of mechanisms occur: with CO as intermediate (Figure 2), when CO_2_ in converted to CO before the methanation [104,105,106,107,108] and direct methanation of CO_2_ (Figure 2) without forming CO as intermediate [109,110].

### 3.1. Methanation of CO_2_ with CO as Intermediate

As a function of the nature and history of catalysts and their reaction conditions, this reaction mechanism is difficult to establish, taking into account that in the literature on CO_2_ methanation, it is believed [104,105,106,107,108,111] that carbon monoxide is a critical intermediate in carbon dioxide methanation. More specifically, the methanation of CO_2_ consists of the reduction of CO_2_ to CO: CO_2_ + H_2_ ↔ CO + H_2_O followed by the conversion of CO into methane (or other alkanes); CO + 3H_2_ ↔ CH_4_ + H_2_O. In this case, the mechanism is identical to that for CO methanation [112,113]. Because the equilibrium for CO_2_ + H_2_ ↔ CO + H_2_O is somewhat unfavorable at the reaction temperatures (200–400 °C), it can be noted that this reaction path is unlikely. One way to circumvent this difficulty is to require that at these temperatures the methanation of carbon monoxide: CO + 3H_2_ ↔ CH_4_ + H_2_O, proceed at significantly faster rates than carbon monoxide production. If carbon monoxide were consumed as rapidly as it forms, no carbon monoxide would be observed in the reactor exit stream [114]. Another mechanism, first suggested by Doehlemann [115] in 1938 and subsequently by Kul’kowa and coworkers [116,117], was also described by Wagner [118]. In this alternative mechanism [118], CO_2_ dissociates into CO_(ad)_ and O_(ad)_, (CO_2__(ad)_ → CO_(ad)_ + O_(ad)_) and the adsorbed oxygen atoms react with molecular hydrogen in a single step (H_2(g)_ + O_(ad)_ → H_2_O_(ad)_), forming water, while the adsorbed CO is transformed into methane (CO_(ad)_ → CH_4_). However, this mechanism assumes that the H_2(g)_ + O_(ad)_ → H_2_O_(ad)_ step is rate-controlling of CO_2_ methanation. In the methanation of CO_2_ on Rh/Al_2_O_3_ [119], the dissociation of CO_2_ into carbon monoxide and oxygen on the surface of the catalysts was also observed in diffuse reflectance infrared Fourier transform (DRIFT) studies. The Rh–CO (2048 cm^−1^), Rh^3+^–CO (2123 cm^−1^), and Rh–(CO)_2_ (2024 and 2092 cm^−1^) bands confirmed the formation of CO_ads_. CO_2_ adsorbed as Rh–(CO)_2_ and CO associated with oxidized Rh are the most hydrogen-reactive species. The presence of CO as a key intermediate in the methanation of CO_2_ was proved by steady-state transient (SST) studies on a Ru/TiO_2_ catalyst [107]. However, at the interface between the metal and support, the presence of formate as a result of reaction with carbonate species was also observed, as an intermediate for the formation of CO. Single crystals of Ni have been suggested as a model catalyst for CO_2_ methanation [120,121]. The dissociation of CO_2_ on Ni(100) [106] proved that CO_2_ is first converted to CO and subsequently to carbon before hydrogenation. The authors observed that the activation energy and reaction rate for CO_2_ methanation are very close to the values for the formation of CH_4_ (88.7 kJ·mol^−1^) from CO (72.8–82.4 kJ·mol^−1^) under identical reaction conditions. It is known also that the formed CO is then dissociated into C and O atoms on the metal sites before being further hydrogenated into methane by the dissociated H_2_ that remains on the metal particles [105,122]. When a Ni(111) surface was tested using atom superposition and electron delocalization-molecular orbital theory [123], the dissociation of CO was found to be the rate-determining step. However, the elementary steps of the CO_2_ methanation reaction consisted of two mechanisms, carbon formation and carbon methanation. For the first mechanism, the activation energies were calculated to be 1.27 eV (~120 kJ^−1^) for CO_2_ dissociation, 2.97 eV (~290 kJ·mol^−1^) for CO dissociation, and 1.93 eV (~190 kJ·mol^−1^) for 2CO dissociation. For the carbon methanation mechanism, the following activation energies were reported: 0.72 eV (~72 kJ·mol^−1^) for methylidyne, 0.52 eV (~50 kJ·mol^−1^) for methylene, and 0.50 eV (~48 kJ·mol^−1^) for methane [123].

### 3.2. Methanation of CO_2_ without CO as Intermediate

Elsewhere, the direct methanation of CO_2_ without CO as an intermediate has also been reported [109,110]. An alternative mechanism for CO_2_ methanation on various catalysts consists of the methanation of CO_2_ through carbonates and formates, which are directly hydrogenated into CH_4_ [107,108,124,125,126,127]. The CO_2_ is first dissociated into CO directly [128,129,130]. CO_2_ methanation on Pd–MgO/SiO_2_ catalysts [126] has shown that the MgO support initiates the reaction. In this case, magnesium carbonate species were observed on the surface of the catalysts. Pd, the active phase of the catalysts, dissociates molecular hydrogen and promotes the hydrogenation of the carbonates and residual carbon atoms [131]. These results show the synergy between the basic support and the active phase. CO_2_, which is an acidic molecule, is activated by the basic sites of the MgO support to form magnesium carbonate, while the metallic sites of Pd dissociate the hydrogen. However, methoxy groups were also observed in the CO methanation mechanism [132]. A Ni/CeO_2_ catalyst showed the highest CO_2_ conversions at lower temperatures with CH_4_ selectivities very close to 100% [133,134,135]. The better performance of CeO_2_ support was attributed to its higher ability to adsorb CO_2_ molecules, followed by its ability to reduce the molecules into CO and then convert the CO into CH_4_. Temperature-programmed reduction (TPR) experiments on Ru catalysts [59] have shown that CO + H_2_ reacts to produce CO_2_ and water, without the production of methane; therefore, gaseous phase CO is not a reaction intermediate during CO_2_ methanation with Ce_0.95_Ru_0.05_O_2_ catalyst [59]. Moreover, CO_2_ was converted directly into methane, without CO as intermediate when nanoporous gallium oxide was used in the photocatalytic conversion of carbon dioxide into methane [136].

## 4. Catalytic Conversion of CO_2_ to CH_4_

The Sabatier reaction, discovered in 1902 by Sabatier and Senderens [137], consists of the catalytic hydrogenation of carbon dioxide to methane. This methanation reaction was investigated by NASA as a necessity for reclaiming oxygen within closed-cycle life support systems. In this case, CO_2_ from the cabin atmosphere is transformed into water vapor, which is electrolyzed and used to return oxygen to the cabin, in addition to one part of hydrogen, as required by the Sabatier reaction. The other part of hydrogen is provided from the electrolysis of stored water, which produces breathable oxygen as a by-product, reducing the proportion of available carbon dioxide that must be reacted and assuring excess carbon dioxide in the feed mixture. In the 1970s, the direct hydrogenation of highly thermodynamically stable CO_2_ attracted significant attention for the production of substitute natural gas (SNG), due to the shortage of natural gas at that time [138]. The ever-increasing demand for natural gas as a fuel and raw material has stimulated renewed efforts to find other means of methane production. With mounting evidence of an “energy crisis” upon us, alternative approaches such as catalytically synthesizing methane from hydrogen and carbon dioxide continue to meet with increased promise for development.

The CO_2_ methanation reaction is reversible and highly exothermic CO_2(g)_ + 4H_2(g)_ → CH_4(g)_ + 2H_2_O_(l)_, ΔH° = −259.9 kJ·mol^−1^, ΔG° = −132.4 kJ·mol^−1^. Because the highly oxidized CO_2_ molecule is highly thermodynamically stable, this compound is not reactive. In this case, CO_2_ methanation requires high-energy substances and entails an eight-electron process with kinetic limitations [131,139]. However, when a reactive catalyst is used, the reaction takes place at low temperatures with high yields and selectivities [131,139]. Porous materials such as microporous zeolite and mesoporous materials have been used for CO_2_ hydrogenation; even so, CO_2_ methanation over zeolites has been investigated less extensively than the hydrogenation of CO. The active phase deposited on the support has been investigated using a number of catalytic systems.

The catalysts used in CO_2_ methanation include the transition elements Ni [106,122,127,140,141,142,143,144,145,146], Pd [147], Pt [148], Co [149,150,151], Rh [66,152,153,154], Mg [155], Zn [156], Zr [157,158], Sn [159], U [160], Ta [161], Nb [162], Cr [163], Ir [164,165], Cu [166], Ag [166], V [167], W [163,168,169,170], Mo [171,172,173], Mn [174,175], Ti [176,177], Fe [72,178,179,180,181]. Excellent reviews of these reports have also been published. Among these metals, Ni, Ru and Rh have been the most effective in this reaction [59,71,104,182,183,184,185]. Ru and Rh have been reported as the most selective toward methane [59,104,184], while Ni has been the most-studied catalyst [71,182] because it presents high activity and selectivity, is cheaper, and hence more interesting from a commercial perspective. Ni has been dispersed on several support types with acidic, basic or neutral sites; in these studies, it was concluded that the activity, selectivity and stability of catalysts made with Ni is determined by the nature of the support. Different interactions can be established between the metal and the support, and these differences influence the catalytic properties of the active metal sites [182]. In spite of the large number of studies performed on Ni-supported catalysts, Ni/Al_2_O_3_ is the best-known catalyst for industrial CO_2_ methanation applications worldwide and it has been commercialized by Evonik, Johnson Matthey, Topsoe and Clariant-Sud Chemie.

The Raney catalysts are very well known in the hydrogenation industry and seem to present high reactivity and selectivity during CO_2_ methanation [186]. Both of these properties have been attributed to the catalyst’s high surface area and structural/thermal properties. The amount of Ni used is also very important because Ni leads to higher methane selectivity [187]. The high activity of Ni/Al_2_O_3_ catalysts in such applications is partially attributed to the presence of a nickel aluminate spinel phase, located at the metal-support interface, which is thought to stabilize the metal particles. Concurrently, it is believed that the formation of a nickel aluminate spinel phase also results in inefficiencies when using Ni-based catalysts [188,189]. Ni/Al_2_O_3_ catalysts prepared through impregnation delivered a rapid deactivation process during an exothermic methanation reaction, resulting in the sintering of Ni particles and severe carbon deposition [190]. Furthermore, the most commonly reported problem associated with Ni-based catalysts is deactivation at low temperatures, due to the interaction of the metal particles with CO and the formation of mobile/volatile nickel carbonyls that lead to the sintering of the metal particles [191,192]. To overcome the problems associated with deactivation and sintering, several solutions have been proposed. As it is commonly known in catalysis chemistry, the addition of a second metal such La, Ce, Sm, Fe, Mg, Y, Pt, Ru, Rh and/or the use of a porous support such as a zeolite or a mesoporous material can be used to inhibit metal sintering [193,194,195,196]. The catalyst containing nanoporous solids is very selective to methane without CO formation. The presence of either the second metal species or the porous structure of the support, which provides a high surface area, can prevent sintering and increase the dispersion of metal particles. For example, when using Al_2_O_3_ or SiO_2_ supports with Ni or Ru metal, the addition of CeO_2_ improves the activity of the system for CO_2_ methanation [134,197,198]. The high activity and selectivity promoted by CeO_2_ have been attributed to its high capacity for metal dispersion and to its propensity to create oxygen vacancies, which promote the reduction of CO_2_ into CO prior to the hydrogenation to CH_4_ [59,129,133,134,135,197,198,199,200].

When using porous surfaces with high surface areas, the interaction between the nickel and its support is a crucial factor, determining the catalytic performance of the system with respect to its activity and selectivity for CO_2_ methanation [145]. Due to the significant ability of the support to disperse the active phase, the preparation of highly dispersed metal-supported catalysts has been the focus of a variety of investigations. Reports have shown that a mesoporous nickel–alumina xerogel [72] can serve as an efficient catalyst in several reactions, due to the well-developed mesoporosity of the support and its ability to finely disperse nickel species [201]. Mesoporous nickel (35 wt %)–metal (M = Fe, Zr, Ni, Y, and Mg) (5 wt %)–alumina xerogel catalysts with a different second metal were tested for use in CO_2_ methanation. In carbon dioxide methanation reaction, the yield of CH_4_ decreased in the order of 35Ni5Fe > 35Ni5Zr > 35Ni5Ni > 35Ni5Y > 35Ni5Mg. The identity of the second metal species influenced the CO_2_ methanation process. The most active and selective mesoporous catalyst was 35Ni5Fe; this catalyst possesses a weak metal support interaction that is closely related to the CO dissociation energy. Fe was found to be the optimal second metal in the CO_2_ methanation reaction. Fe can modify the size of metallic Ni particles and the reducibility of the Ni which leads to changes of the local electron density. These results show that the metal-support interaction, the CO dissociation energy and the pore volume influenced the CO_2_ conversion. The use of a Ni/RHA–Al_2_O_3_ catalyst with a mesoporous structure and high surface area exhibited favorable catalytic activity [145]. Nanocrystallites of nickel oxide, such as NiO and NiAl_2_O_4_, are formed with high dispersion on the surface, suggesting a strong interaction between the metal and oxide. The catalytic activity of Ni/RHA–Al_2_O_3_ is better than that of Ni/SiO_2_–Al_2_O_3_ due to its enhanced metal dispersion ability and higher chemical reaction rate. At 500 °C, a conversion of 58% and a methane selectivity of 90% were obtained [145]. On 3 w% Ni/MCM-41 catalysts, [146] a high selectivity (96.0%) were achieved at a space velocity of 5760 kg^−1^·h^−1^, which was superior to the results obtained with a Ni/SiO_2_ catalyst and comparable to that of a Ru/SiO_2_ catalyst [106,142,202]. The high selectivity was maintained at a higher reaction temperature (400 °C). The best activity and selectivity for CO_2_ methanation when using a Ni/MCM-41 mesoporous catalyst is attributed to the highly dispersed Ni^0^ at 700 °C, as obtained on a surface [146]. A mesoporous catalyst of Ni/SiO_2_ was reported to be more active than Ni/Al_2_O_3_ in CO_2_ methanation [203], although other papers have reported very high activities of highly loaded Ni/Al_2_O_3_ catalysts [204], which even can be increased by Fe-doping [205]. Ni/ZrO_2_ catalysts doped with Ce or Sm cations [196] exhibit higher catalytic activity for CO_2_ methanation. This behavior can be ascribed to a synergistic effect between the surface area and doping with rare earth elements. Up to 280 °C, the Co/KIT-6 mesoporous catalyst exhibits a higher CO_2_ methanation activity, with a conversion of 49% and a methane selectivity of 100% [151]. Its high methane selectivity has been attributed to the high degree of dispersion obtained on its large surface area as well as its highly ordered bicontinuous mesoporous structure.

Zeolites are an attractive support material due to their high thermal stability and large surface area. Encouraging results have been obtained for the methanation of CO [206,207,208,209,210,211] and CO_2_ on zeolites [71,212]. High activity and selectivity during CO_2_ methanation was reported for Y zeolite, which possesses a higher mesoporosity; in this case, the zeolite structure accounts for the improved kinetics [213]. Of the Ru/HZSM-5 and Ru/SiO_2_ catalysts, the former is more selective for CH_4_. This behavior can be explained by the higher amount of CO_2_ that is able to react with the OH groups of the zeolite, in addition to the stronger interaction between the metal and the support. Fourier Transform Infrared (FTIR) [214] on Ru/zeolite have shown that CO_2_ methanation takes place by the dissociative adsorption of CO to form CO*ad* and O*ad*, followed by conversion into CH_4_ and H_2_O. The hydrogenation of CO_2_ has been investigated in a dielectric barrier discharge (DBD) plasma reactor packed with 10 wt % Ni/zeolite pellets within a temperature range of 180–360 °C [215]. In this case, less than 15% CO_2_ conversion was observed in the catalytic system; in comparison, the non-thermal plasma created in the catalyst bed increased the conversion of CO_2_ by more than 95%. These results suggest that the formation of a reactive species in the plasma reactor can speed up the rate-determining step of catalytic hydrogenation. The high conversion of CO_2_ was attributed to the smaller Ni particles and their uniform dispersal over the zeolite after the plasma reaction. The hydrogenation of CO_2_ involves the dissociation of CO_2_ to C-O and O on the active site of Ni/zeolite [216,217]. Ni/USY and NiCe/USY zeolite catalysts [71] exhibit high conversion rates and selectivity for methane production during CO_2_ methanation. When using a large amount of Ni with a high proportion of Ni^0^, the conversion is favored. The presence of CeO_2_ after reduction might promote the activation of CO_2_ into CO. The zeolite catalyst shows no deactivation or sintering of the Ni metal particles.

These results show that the performance of nanoporous catalyst materials depends on a variety of parameters, including particle size and shape, amount of metal, nature of the metal and the support, and evolution of the catalyst surface during thermal treatment. The role of a large pore/surface is crucial to ensure enhanced metal dispersion, high diffusivity and longer catalyst lifetime. An important aspect is the use of nanopores/nanocavities, which could favor the local increase of CO_2_ concentration—nanoconfinement—and thus the consecutive conversion of intermediates with formation of CH_4_.

## 5. Photocatalytic Reduction of CO_2_ into CH_4_

To replace fossil fuels with fuels derived from recycled CO_2_, photovoltaics might be able to generate the energy necessary to produce these fuels. The photocatalytic reduction of CO_2_ with H_2_O, which is an important reaction, especially as a means of carrying out artificial photosynthesis, has been attempted in light of the importance of carbon storage [218].

The first study carried out on the photocatalytic reduction of CO_2_ with H_2_O used TiO_2_ and SrTiO_3_ as photocatalysts [219] and yielded HCOOH, HCHO as the principal product, and CH_3_OH and CH_4_ in trace amounts. Furthermore, various semiconductors, such as tungsten trioxide (WO_3_), titanium dioxide (TiO_2_), zinc oxide (ZnO), cadmium sulfide (CdS), gallium phosphide (GaP), and silicon carbide (SiC) activated by both xenon- and mercury-lamp irradiation, have been used for such purposes [98]. Photocatalytic CO_2_ reduction is more difficult to perform and delivers with a lower efficiency [220,221,222,223] due to the associated thermodynamics and kinetics. The reduction of CO_2_ by H_2_O to obtain methane is a highly endothermic process (±802 kJ·mol^−1^) and requires a significant amount of energy. This energy is later released during the oxidation of the fuel. The reaction mechanisms for CO_2_ reduction necessitate either the consecutive or simultaneous transfer of electrons and photons to the CO_2_. CO_2_ reduction utilizes electrons with higher reduction potential. The best-known catalysts for the photocatalytic reduction of CO_2_ with water are TiO_2_. It should be noted that TiO_2_ is not photoresponsive under visible light irradiation, limiting its use as a photocatalyst. To obtain a photoresponse in the visible region, TiO_2_ catalysts must be doped with metals [224,225,226], non-metals and oxygen vacancies [227,228] or noble metal doping must be used [229,230]. However, it has been observed that for reactions carried out in water, the doped photocatalysts present photoconversion but also lead to dopant metal leaching and catalyst deactivation. Excellent articles on these topics have also been published [98,218,231,232,233,234,235,236,237,238,239,240]. It has further been observed that the use of nanoparticle semiconductors can provide a higher activity for CO_2_ reduction compared to the corresponding bulk semiconductor [234]. It has been observed that the photocatalytic activity increased with the decreasing diameter of the TiO_2_ particles, although sunlight radiation was increasingly less utilized. However, when Pt was added to the TiO_2_, a “short-circuited photoelectrochemical cell” providing both oxidizing and reducing sites for the reaction developed [234]. Often, a clear distinction between the mechanisms of the photoreduction and photoelectrochemical reduction of CO_2_ is not possible, particularly when using metal-doped semiconductor materials. The reaction mechanism in the photoreduction of CO_2_ involves two important species, the hydrogen atom and carbon dioxide anion radical, which is produced by electron transfer from the conduction band.

Some investigations have pursued the development of highly dispersed transition metal oxides, such as Ti, V, Cr, Mo, inside micro- and mesoporous materials as nanophotocatalysts. Zeolites contain only isolated metal ions in their framework structures. These nanophotocatalysts can be excited under UV irradiation to form the corresponding charge-transfer excited states involved in electron transfer. The reactivities of charge-transfer excited states, *i.e.*, electron-hole pair states, are responsible for photocatalytic reactions such as the reduction of CO_2_ with H_2_O to produce CH_4_ and CH_3_OH [218,241,242,243]. However, in the photocatalytic reaction, CO_2_ adsorbs onto the surface of the photocatalyst due to its microporosity, which permits the concentration of the substrate near reactive sites to increase and decreases the activation energy of the process. The use of microporous zeolites permits increased CO_2_ adsorption and can introduce diffusion through the pores. It has also been reported that CO, CH_4_, H_2_, and higher hydrocarbons can be produced during the photocatalytic reduction of CO_2_ on TiO_2_ surfaces in the presence of gaseous H_2_O [244]. In contrast, it is known that the octahedrally coordinated bulk TiO_2_ photocatalyst is not selective for the photocatalytic reduction of CO_2_ with gaseous H_2_O. Instead, the selectivity of the photocatalytic reaction is favored on the tetrahedrally coordinated titanium dioxide photocatalysts of the silica matrix when the activity and selectivity are favored, leading to the significant formation of CH_3_OH [245,246,247,248,249]. Ti-containing micro- and mesoporous zeolites have exhibited efficient and selective photocatalytic reactivity for the reduction of CO_2_ with H_2_O using UV radiation. The hydrophobicity and hydrophilicity of zeolite are additional parameters that affect the activity and selectivity of the photocatalytic reduction of CO_2_ with H_2_O when attempting to produce CH_4_ and CH_3_OH through this reaction [218]. It has been shown that the competition between CH_3_OH and CH_4_ is governed by the hydrophobicity/hydrophilicity of β-zeolite, as studied for the photocatalytic reduction of CO_2_ with H_2_O at 50 °C. The activity of hydrophilic Ti–β–OH was found to be higher than that for hydrophobic Ti–β–F. However, the selectivity for the formation of CH_3_OH from Ti–β–F (41%) was higher than that from Ti–β–OH (11%). Ti–β–OH exhibited a higher reactivity compared to Ti–β–F, although its selectivity was different. On the hydrophilic Ti–β–OH zeolite, the selectivity for the formation of methane was higher than that with TS–1, Ti–β–F and P25. The selectivity for methane followed the order Ti–β–OH > TS–1 > Ti–β–F > P25. The higher activity of Ti–β–OH toward methane formation has been explained by the higher concentration of charge-transfer excited complexes. Furthermore, it has been shown previously [245,246] that the competitive interaction of CO_2_ and H_2_O molecules with the charge-transfer excited state of the tetrahedral titanium oxide species results in the formation of C radicals, H atoms, and OH radicals on the surface, while CH_4_ and CH_3_OH are formed by the reaction of C radicals with H atoms and OH radicals [245,246]. When using hydrophilic Ti–β–OH, the concentration of H_2_O is significantly higher than that obtained with hydrophobic Ti–β–F, leading to a higher selectivity for the formation of methane. A higher selectivity for methane was also observed on Ti/FSM–16 [218], while the use of fluorinated Ti/FSM–16 led to the higher formation of CH_3_–OH, even though the formation of methane was a major reaction pathway in both cases.

**Figure 3:**
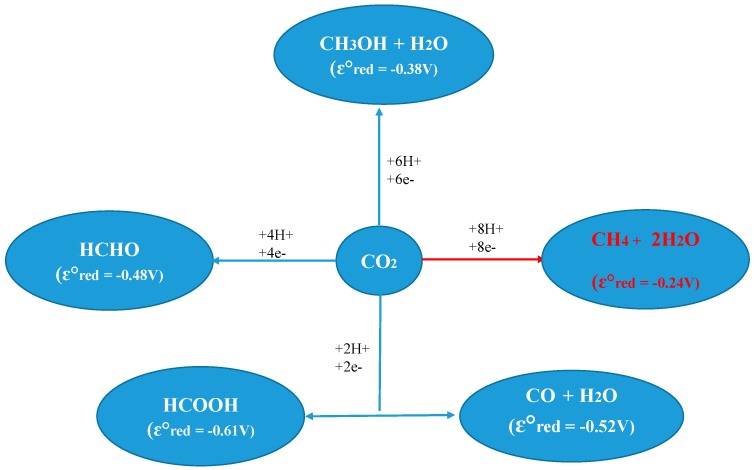
Photocatalytic reduction of CO_2_ to fuels [98,220,221,232].

A Ti/Y zeolite containing tetrahedral isolated sites was tested for the photocatalytic reduction of CO_2_ in the presence of water using UV light from a high-pressure Hg lamp (>280 nm) at 55 °C [246,247]. The tetrahedrally arranged Ti sites formed methanol with methane as the major product, while the Pt/Ti–Y zeolite selectively formed methane. In this case, CO was an apparent intermediate for the reduction of CO_2_, although H_2_ was not. It is possible that the water was oxidized to OH and H^+^ and that the Pt sites likely worked to suppress charge recombination [246]. Similarly, Ti–MCM–48 produced both methane and methanol, although Pt/Ti–MCM–48 was found to be the most active catalyst and selectively formed methane [247]. Ti–SBA–15 also produced methane and methanol, where methane was the major product [248,249,250]. The methane yield of highly dispersed tetrahedral isolated sites of titanium nanophotocatalyst was increased 300 times as compared to crystalline TiO_2_. The activity was attributed to photo-excited Ti centers generated by a Ligand to Metal Charge Transfer transition (Ti^+IV^ − O^−II^→Ti^+III^ − O^−I^) upon light absorption [248]*.* Pt-loading on the Ti-containing zeolite catalyst leads to an efficient quenching of the photoluminescence, accompanied by the shortening of its lifetime [235]. The reaction mechanism in the photocatalytic reduction of CO_2_ with H_2_O on the highly dispersed Ti oxide catalyst can be proposed to occur as follows: CO_2_ and H_2_O molecules interact with the excited state of the photo-induced (Ti^3+^ − O^−^)^∗^ species, and the reduction of CO_2_ and the decomposition of H_2_O proceeds competitively. Furthermore, H atoms and OH• radicals are formed from H_2_O and these radicals react with the carbon species formed from CO_2_ to produce CH_4_ and CH_3_OH [235]. The reaction mechanism in the photoreduction of CO_2_ involves two important species, the carbon dioxide anion radical (CO_2_ + e^−^ → CO_2_) and hydrogen atom (H^+^ + e^−^ → H), which is produced by electron transfer from the conduction band. Multielectron reactions compete with these reactions (Figure 3). As observed from Figure 3, the thermodynamic potential of CO_2_ reduction decreases even if several electrons and protons could be simultaneously transferred in pairs to CO_2_. It is kinetically unfeasible for the reactions where all protons and electrons needed to form CH_4_ and H_2_O are transferred in a single step. This suggests that the appropriate photocatalyst should have photocatalytic centers in which sites transferring electrons are close enough to other sites acting as acids and transferring at least one proton according to Figure 3.

In this case an efficient photocatalyst should have appropriate catalytic sites where chemical redox processes leading to the desired products could take place with low activation barriers. The basic sites and porosity can play a favorable role in the photocatalytic process by increasing the concentration of the substrate near the reactive sites and by decreasing the activation energy of the process. It must be noted that the solubility of CO_2_ in water is low, and the CO_2_ photoreduction reaction competes with H_2_O_2_ and H_2_ formation, as follows:

2H_2_O + 4h^+^ → O_2_ + 4H^+^ (27)

4H^+^ + 4e^−^ → 2H_2_ (28)

O_2_ + 2H^+^ + 2 e^−^ → H_2_O_2_ (29)

Methanol and formaldehyde are the easier products of CO_2_ reduction in water solution. If the water is replaced by the other reductants such as low-polarity solvents or low-dielectric constant solvents, CO is formed as the major product. CO_2_ anion radicals are strongly adsorbed on the surface through the carbon atom of another CO_2_ anion radical because these radicals are not well dissolved in low-polarity solvents [98]. When a high-dielectric constant solvent is used, formic acid is formed as a major product, because the CO_2_ anion radicals can be greatly stabilized by the solvent, resulting in weak interactions with the photocatalyst surface and the carbon atom of the radical reacts with a proton to produce formic acid. In order to the increase water solubility, basic pH values are necessary, but this converts CO_2_ into CO_3_^2−^ or HCO_3_^−^ that are more stable and more difficult to reduce than CO_2_ itself. Consequently, it could be of interest to study other solvents or to perform CO_2_ reduction in the gas phase [98].

## 6. Conclusions

This review summarizes recent studies performed on the methanation of CO_2_ on nanoporous materials. Although our knowledge of nanoporous materials is relatively well developed, several challenges remain with respect to their use in methanation processes. This review has presented an extensive series of investigations of CO_2_ methanation over various catalysts. The best results concerning the activity/selectivity and the lifetime of catalysts have been obtained for Ru and Rh. However, the high costs associated with these catalysts impedes their use at large scale, such as commercial applications. Ni is the best alternative candidate due to its high selectivity for methane production. The role of the support dominates the catalyst design in terms of developing an efficient methanation catalyst, specifically with respect to ensuring enhanced metal dispersion and a long catalyst lifetime. For this reason, nanoporous materials provide the best supports for Ni (Ru, Rh, Co, Fe) particles because they can prevent sintering and deactivation through coking, which otherwise blocks the metal surface as carbon accumulates. Because the methanation reaction is extremely exothermic, the excessive heat of reaction induces metal sintering, which lowers the overall metal surface area and leads to the poor activity observed for the classical supports. Thus, it is necessary that we develop an efficient, low-temperature methanation catalyst with high thermal stability and coke formation resistance.

Many investigations have focused on CO_2_ methanation, but significant effort must still be made in the coming years to understand the fundamental reaction mechanisms in order to improve the activity and the selectivity of catalysts for methane. The results of our study have demonstrated that there remains a lack of a conceptual framework regarding nanoporous catalysts and that we must enhance our understanding of the nanoarchitecture of the active sites to improve the catalytic and photocatalytic selectivity of nanomaterials toward methane. Furthermore, the results show that the use of highly dispersed small particles on supports with high surface areas and highly dispersed tetrahedrally coordinated sites serve as the active sites for high methane selectivity. The use of nanoporous catalysts is one of the most promising approaches in the design of efficient local structures for catalysts at the molecular level, toward the development of effective methanation catalysts.
